# The effect of the art therapy interventions to alleviate depression symptoms among children and adolescents: a systematic review and meta-analysis

**DOI:** 10.1016/j.clinsp.2025.100683

**Published:** 2025-05-13

**Authors:** Bo Zhang, Lifang Yang, Wen Sun, Peng Xu, Hui Ma, Azizah binti Abdullah

**Affiliations:** a Guizhou Equipment Manufacturing Polytechnic, Guiyang, PR China; b Universiti Utara Malaysia, Kedah, Malaysia; c Guizhou Medical University, Guiyang, PR China; d Hainan Provincial Anning Hospital, Hainan, PR China

**Keywords:** Adolescents, Children, Depression, Art therapy

## Abstract

**Background:**

Existing systematic reviews provide evidence that art therapy is an effective intervention for reducing symptoms of depression in children and adolescents. This study aims to systematically review and quantify the impact of art-based interventions on depressive symptoms in this demographic through meta-analysis.

**Method:**

A systematic search of databases and journals identified eligible studies for inclusion in a meta-analysis examining the efficacy of art therapy interventions on depressive symptom reduction. A random-effects meta-analysis was conducted using the Standardized Mean Difference (SMD).

**Results:**

The meta-analysis of twelve (randomized) controlled trials revealed a significant effect (SMD = -0.72; 95 % CI [-1.28, -0.16], *p* = 0.01), indicating that children and adolescents (*n* = 333) who received art therapy experienced a more significant reduction in depressive symptoms compared to those (*n* = 344) in the control groups.

**Limitations:**

The existing evidence is limited to studies with low statistical power and methodological quality. Furthermore, the included studies were highly heterogeneous in terms of the intervention type, measurement tools, participant samples, and study designs.

**Conclusions:**

The findings suggest that art-based interventions can be effective in reducing depressive symptoms in children and adolescents. However, future research requires more methodologically rigorous and well-controlled studies to strengthen the evidence base.

## Highlight

• Systematic review & meta-analysis of art therapy for child & adolescent depression.
• Art therapy significantly reduces depressive symptoms (SMD = −0.72, *p* = 0.01).
• Visual arts, music, drama, and other interventions are effective therapeutic tools.

## Introduction

In recent years, mental health issues among children and adolescents have increasingly become a concern. According to a 2022 World Health Organization report, approximately 14 % of adolescents aged 10‒19 worldwide are estimated to have a diagnosed mental disorder^1^ Depression is a prevalent issue in this age group, with a global risk of clinical depression affecting approximately 34 % of adolescents aged 10‒19^2^ The impact of depression extends beyond psychological well-being, negatively influencing academic performance, interpersonal relationships, and overall functionality^3^ Up to 67 % of adolescents exhibiting depressive symptoms may develop clinical depression or anxiety disorders in adulthood, along with an increased risk of suicide. This indicates that depressive symptoms during adolescence are strong predictors of future mental disorders^4^ Therefore, the early detection and treatment of depression in children and adolescents are crucial for promoting the physical and mental well-being of this population.

Over the past few decades, the primary interventions for adolescent depression have encompassed pharmacological treatments, psychosocial therapies, and adjunct modalities. Firstly, pharmacological interventions involving antidepressant medications can effectively alleviate depressive symptoms in youths; however, they may not necessarily enhance coping abilities^5^ The safety of antidepressants remains contentious, with some studies suggesting an increased risk of suicidal ideation among pediatric populations, coupled with issues of poor adherence and numerous adverse effects^6^^,^^7^ Secondly, psychosocial interventions, such as cognitive-behavioral therapy, interpersonal therapy, and family therapy, are widely employed in the psychological management of adolescent depression^8^ Although these therapies are generally regarded as beneficial, considerable evidence suggests that their underlying premise – cognition influencing emotions and behaviors – may not be suitable for all adolescents, particularly those who struggle with articulating their experiences and feelings^9^ The efficacy of these interventions is challenging to measure and may produce adverse effects, rendering this perspective questionable^10^ Thirdly, research on adjunct therapies, such as exercise and art therapy, has garnered attention as potentially safer and non-invasive modalities, demonstrating improvements in depressive mood and mental well-being among youths^11^ However, most studies in this domain have small sample sizes, lack control groups, and the long-term sustainability of therapeutic effects remains to be confirmed^12^

Art therapy, a non-pharmacological complementary and alternative medical approach, utilizes the creative art process to promote individuals' physical and mental well-being^13^ Within this modality, art therapists guide individuals through a therapeutic process involving interaction with art materials, fostering a playful and safe environment conducive to positive change in psychosocial dynamics. This process enables children and adolescents to tap into emotions that are often inexpressible through verbal means^14^ The practice of self-expression may foster a sense of control, self-efficacy, and self-discovery, potentially providing clinicians with an alternative approach to address psychosocial issues compared to conventional therapies^15^ Empirical evidence suggests that AT may alleviate depressive symptoms in young individuals. A meta-analysis, for instance, demonstrated that music therapy significantly ameliorates internalizing problems, such as depression and anxiety, in children and adolescents^16^ Additionally, a systematic review suggests, albeit with limited evidence, that music therapy is an effective treatment for reducing the severity of depressive symptoms in this population^16^ However, more high-quality randomized controlled trials are needed to address the methodological limitations of current research^17^

Although existing evidence indicates the benefits of music therapy for depressive symptoms, the impact of art therapy as a whole on depression remains less clear. The measurement of impact may vary due to the heterogeneity of art forms, including visual arts, drawing, painting, music, dance, drama, and writing. This variability stems from the distinct psychodynamic processes and methodologies inherent to each creative art therapy modality^18^ It is important to note that while meta-analyses have demonstrated the efficacy of music therapy, similar comprehensive studies encompassing the broader range of art therapy interventions are lacking, indicating a need for further research to understand the extent of their effectiveness on depressive symptoms fully. This study aims to address the existing research gap by conducting a systematic review and meta-analysis of clinical trials pertaining to the comprehensive application of art therapy. The objective is to synthesize the current evidence and evaluate the true efficacy of art therapy interventions in mitigating depressive symptoms among children and adolescents.

## Method

This study strictly adhered to the guidelines outlined in the Preferred Reporting Items for Systematic Reviews and Meta-Analyses (PRISMA) statement^19^ The study protocol has been registered in the International Prospective Register of Systematic Reviews (PROSPERO) under the registration number CRD42023457357.

### *Search strategy*

The search strategy was designed, and the initial literature search was conducted independently by two authors across PubMed, the Cochrane Central Register of Controlled Trials, PsycINFO, Embase, Web of Science, Scopus, and the Chinese databases Wanfang Data and China National Knowledge Infrastructure. The search aimed to identify relevant Randomized Controlled Trials (RCTs) and non-randomized controlled trials from inception to October 11, 2023, without year restrictions but with language limitations to English and Chinese. For instance, in PubMed, The authors combined keyword searches with Medical Subject Headings (MeSH) terms: (depression OR depressive symptoms OR depressive symptom OR symptom, depressive OR emotional depression OR depression, emotional, MeSH terms: depression) AND (music therapy OR visual art* therapy OR dance therapy OR painting therapy OR sculpture therapy OR movement therapy OR poetry therapy OR therapies, art OR art therapies OR drawing therapy OR therapy, MeSH terms: art therapy) AND (randomized controlled trial OR randomized OR placebo). Search terms were adjusted for each database based on their respective subject headings. Additionally, the authors manually scanned the reference lists of relevant prior studies to identify potential studies recursively. All analyses were based on published studies, and no ethical review or patient consent was required^19^

### *Selection criteria*

This study included peer-reviewed Randomized Controlled Trials (RCTs) that evaluated the impact of art therapy interventions on depressive symptoms in children and adolescents (aged ≤ 18-years). To ensure clarity and consistency, the authors established specific inclusion and exclusion criteria. Studies were included if they were peer-reviewed RCTs assessing the effects of art therapy on depressive symptoms in individuals aged 18-years or younger and were published in English or Chinese. Studies were excluded if they were non-randomized studies, observational studies, meta-analyses, reviews, case reports, case series, or letters to the editor. Additionally, studies that did not report depressive symptoms as an outcome or provided insufficient data for meta-analysis were also excluded.

### *Data extraction*

In this study, the authors utilized the EndNote software to integrate the search results and effectively remove duplicate references. Two researchers independently screened the titles and abstracts to exclude studies that did not meet the predefined inclusion criteria. Subsequently, researchers independently performed data extraction, and any disagreements encountered during the data extraction process were also resolved through consensus. The authors extracted the following information from the included clinical trials: first author's name, year of publication, country where the study was conducted, study design, sample sizes of the intervention and control groups, characteristics of the study population, details of the art therapy intervention, duration and frequency of treatment, measurement methods employed, and reported results.

### *Quality assessment*

The methodological quality of the included studies was evaluated using the Delphi scoring criteria, which cover the following aspects: a) Implementation of a standard randomization procedure, b) Allocation concealment, c) Participant blinding, d) Caregiver blinding, e) Outcome assessor blinding, f) Baseline comparability between groups, g) Clear definition of inclusion criteria, h) Presentation of outcome variability, and i) Conduct of intention-to-treat analysis. According to these criteria, a maximum score of 9 was assigned to each study. Studies scoring 7 or above were considered high quality, while those scoring 6 or below were deemed low quality.

### *Statistical analysis*

This meta-analysis was conducted using Review Manager 5.3 and Stata 14 software. Given the variability in participant characteristics and the instruments used to assess depressive symptoms across the included studies, the authors utilized a random-effects model (Hartung-Knapp method) to pool the Hedges' g effect sizes and their 95 % Confidence Intervals. This approach aimed to yield more conservative and broadly applicable summary estimates. The Hedges' g effect sizes were derived from the means, standard deviations, and sample sizes of each intervention group post-treatment or during follow-up periods^20^ A negative effect size signifies that art therapy interventions have an advantage over control groups in reducing depressive symptoms. Heterogeneity among studies was assessed using the Cochran *Q* test, and the Higgins I²statistic was employed to measure the extent of variability attributable to heterogeneity, with I² values of 25 %, 50 %, and 75 % categorized as low, moderate, and high levels, respectively. Publication bias was evaluated with the Egger regression test, where a p-value of <0.10 indicated significant bias. Additionally, sensitivity analyses were conducted by identifying studies with 95 % Confidence Intervals that did not overlap with the summary estimate as outliers. These studies were then excluded, and the effect size was recalculated to test the robustness of the findings.

### *Ethical considerations*

This systematic review and meta-analysis was approved by the Ethics Committee of Guizhou Equipment Manufacturing Polytechnic (Approval nº GEMPC-2024–003). Since this study is based on previously published data and does not involve direct human participants, the requirement for informed consent was waived.

## Result

**Fig. 1:**
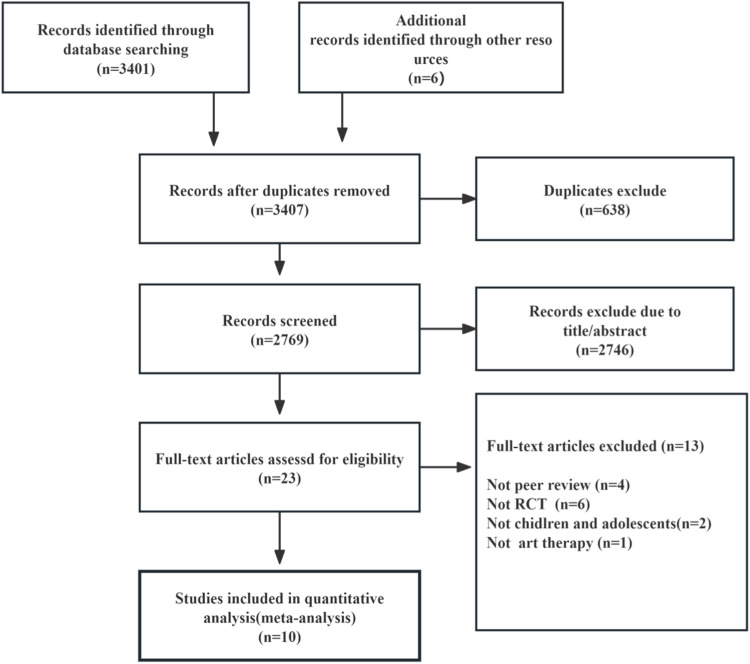
Literature screening flow chart.

As of October 10, 2023, the meta-analysis initially identified 3407 studies through database searches and reference tracking. After deduplication, 2769 studies were screened based on title and abstract, leading to the exclusion of 2746 studies. A full-text review was conducted on the remaining 23 studies, and 12 were subsequently excluded for failing to fulfill the inclusion criteria. Ultimately, 10 controlled trial studies were included in the meta-analysis (Fig. 1)^21^, ^22^, ^23^, ^24^, ^25^, ^26^, ^27^, ^28^, ^29^, ^30^ The total number of participants in the included studies was 657, with 333 in the control groups and 324 in the intervention groups. All included studies were published in English or Chinese (Table 1). Notably, the study by Rezazadeh et al^24^ featured a three-arm randomized controlled trial design. Such a design typically involves a shared control group, and special attention should be paid to the handling of this control group when conducting the meta-analysis. Specifically, while this control group can be compared separately with other experimental groups when pooling results from different studies, care should be taken to avoid duplicating the sample size of this control group when calculating the total sample size, as this could lead to an overestimation of the overall sample size.

**Table 1:** Characteristics of the studies included in the analysis.

1st Aut, year	Country	Study type	Number/ (treated/ control)	Study population	Art therapy characteristics	Control condition	Treatment duration, frequency	Measure	Result	Quality
Zamanifard, 2022	Iran	RCT	20/20	Children aged 8‒12 years with type 1 diabetes who were referred to Imam Reza Clinic of Diabetes in Shiraz.	Virtual directed painting therapy, directed by WhatsApp, with six 2-hour group sessions once a week for six weeks.	Routine care provided by the Clinic of Diabetes, including nutritional, psychological, and exercise counseling.	Six weeks, with one 2-hour session per week.	CDI	*p* < 0.001	Low
Gürcan, 2021	Turkey	RCT	30/30	Hospitalized adolescents with cancer, aged between 12 and 17 years.	Mandala drawing	Routine care only.	Two sessions with an interval of 2 or 3 days.	HADS	*p* < 0.01	High
Wagener, 2012	United States	RCT	21/20	Obese adolescents aged 12‒18 years.	Dance-based exergaming program.	Wait-list control condition	10-week program with three sessions per week.	BASC-2	Clinically significant	High
Porter, 2017	United Kingdom	RCT	50/65	Participants were 8 to 16 years old with social, emotional, behavioral, and developmental difficulties	Music therapy	Usual care	12 weekly sessions, each lasting 30 min.	CES-DC	*p* = 0.006	High
Walsh, 1993	United States	A pretest-posttest repeated-measures design	17/18	Hospitalized suicidal adolescents	Art future-image intervention	Informal recreation	3-hours, in two 1.5-hour sessions	BDI	*p* = 0.58	Low
Cheung, 2018	China, Hong Kong	RCT	30/30	Chinese Pediatric Brain Tumor Survivors	Musical training	Placebo control intervention	Once a week for 45 min for 52 weeks	CES-DC	*p* = 0.005	High
Rezazadeh, 2020	Iran	RCT	40/20	6–12-year-old children with burns	Painting therapy and music therapy	Routine care	10 daily 45-min art therapy sessions	CDI	Painting group *p* < 0.01	High
									Music group *p* < 0.01	
Currie, 2012	Australia	RCT	28/26; 22/22	Reactively aggressive 12–15-year-old males	Group psychotherapy sessions using percussion instruments	Wait-list control condition	Twice a week for 10-weeks	BDI	*p* < 0.05	High
Ran, 2021	China	RCT	22/20	Adolescents with Depression	Painting therapy	Routine antidepressant treatment and care	Eight treatments, Once a week	Childhood Depression Self-Rating Scale (SRSC)	*p* = 0.005	Low
Zhou, 2022	China	RCT	40/40	Children with Emotional Disorders	Painting therapy	Routine care	Three stages, each 10 days, a total of 30 days	SDS	0.000	Low

BDI, Beck Depression Inventory; CDI, Children’s Depression Inventory; CES-D, Center for Epidemiologic Studies Depression; HADS, Hospital Anxiety and Depression Scale; SDS, Self-Rating Depression Scale; BASC-2, The Behavior Assessment System for Children-2; DSRSC, Depression Self-Rating Scale for Children.

### *Study characteristics*

Table 1 provides a summary of the characteristics of the included studies. All 10 studies utilized an RCT design. Participants were children and adolescents, ranging in age from 8 to 18 years, with a variety of conditions, including type 1 diabetes, obesity, cancer, depression, and burn injuries.

The art therapy interventions employed across the studies were diverse, including virtually guided painting, mandala drawing, interactive dance games, music therapy, and drumming group therapy. The control conditions ranged from usual care to waitlist control and placebo control. The duration of interventions varied from 2 sessions to 52 weeks, with 10 to 12 weeks being the most common duration. The session frequency ranged from once a week to thrice a week. The length of each ses3 hoursried from 30 min to 3 hours.

### *Quality of the studies*

Based on the Delphi criteria, six of the studies were deemed to be of high quality,^23^^-^^25^^,^^27^^-^^29^ while four were categorized as low quality^21^^,^^22^^,^^30^ (Table 1).

### Meta-analysis (including overall and subgroup)

#### Overall analysis

**Fig. 2:**
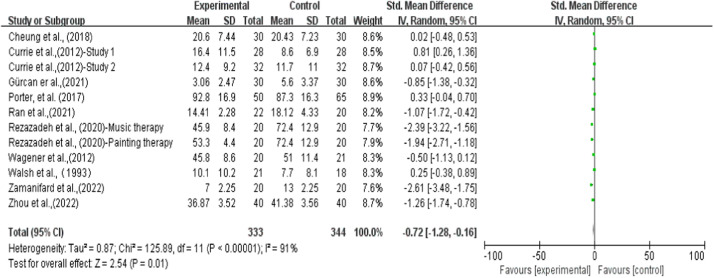
Effects of art therapy on depression.

The effects of art therapy on depressive symptoms were evaluated in 12 RCTs. These 12 RCTs originated from 10 articles, wherein Rezazadeh et al^24^ was a three-group randomized clinical trial that was separated into one painting therapy experiment, and one music therapy experiment, and Currie & Startup^23^ consisted of two separate randomized controlled studies. This meta-analysis included 657 participants, analyzing solely the results from the first measurement after treatment completion. Employing a random-effects model, the meta-analysis results demonstrated that the art therapy group exhibited significantly lower levels of depression compared to the control group (SMD = −0.72; 95 % CI [−1.28, −0.16], *p* = 0.01), albeit with substantial heterogeneity (I² = 91 %). Detailed information can be found in Fig. 2.

#### Subgroup analysis

The results of the subgroup analysis further revealed three main study patterns: painting therapy, music therapy, and dance therapy.

**Fig. 3:**
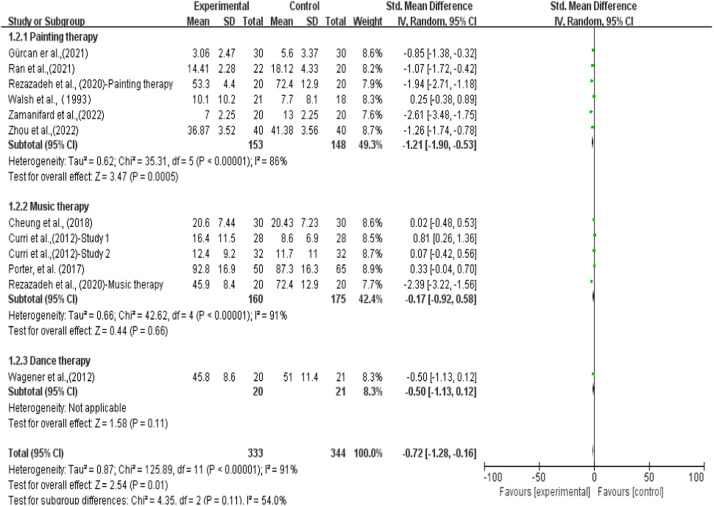
Effects of painting therapy, music therapy and dance therapy to reduce depression.

For the painting therapy subgroup, the data exhibited an SMD of −1.21 (95 % CI [−1.90, −0.53]), indicating a statistically significant overall effect (*Z* = 3.47, *p* < 0.0005). However, considerable heterogeneity was observed, with Tau² = 0.62, Chi² = 35.31, df = 5, I² = 86 %. The music therapy subgroup exhibited an SMD for music therapy was −0.17 (95 % CI [−0.92, 0.58]), indicating a non-significant overall effect (*Z* = 0.44, *p* = 0.66). Significant heterogeneity was also present, with Tau² = 0.66, Chi² = 42.62, df = 4, I² = 91 %. The dance therapy subgroup had an SMD of −0.5 (95 % CI [−1.13, 0.12]). However, as only one study was included, the subgroup analysis was insufficient to draw statistical conclusions (Fig. 3).

The high degree of heterogeneity across studies, with an I² statistic reaching 91 %, indicated substantial variability in outcomes. This variability could be attributed to several factors, including differences in study methodologies, participant demographics, the specific application of art therapy, and the methods used for outcome measurement. Given the significant heterogeneity, a traditional meta-regression analysis was deemed inappropriate due to its requirement for homogeneity across studies to estimate the overall effect size accurately and to explore variable relationships. In the presence of such heterogeneity, a pooled effect size could be misleading. Consequently, to avoid the potential for an ecological fallacy, a meta-regression analysis was not performed.

### *Sensitivity analyses*

To assess the robustness of the present findings, the authors conducted sensitivity analyses by systematically excluding each study and evaluating its impact on the pooled estimate. Notably, the direction and magnitude of the summary estimate remained consistent, irrespective of which specific studies were excluded. This consistency demonstrates the resilience and robustness of the meta-analytic findings (Table 2).

**Table 2:** Sensitivity analysis results.

Study Omitted	Effect Size (95 % CI)	Heterogeneity I²
None (All Studies)	[−1.28, −0.16]	91 %
Cheung et al. (2018)	[−1.40, −0.19]	92 %
Curri et al. (2012) ‒ Study 1	[−1.42, −0.30]	90 %
Curri et al. (2012) ‒ Study 2	[−1.41, −0.91]	92 %
Gürcan et al. (2021)	[−1.32, −0.10]	92 %
Porter et al. (2017)	[−1.41, −0.23]	91 %
Ran et al. (2021)	[−1.28, −0.10]	92 %
Rezazadeh et al. (2020) ‒ Music therapy	[−1.12, −0.04]	90 %
Rezazadeh et al. (2020) ‒ Painting therapy	[−1.17, −0.05]	91 %
Wagener et al. (2012)	[−1.35, −0.14]	92 %
Walsh et al. (1993)	[−1.40, −0.22]	92 %
Zamanifard et al. (2022)	[−1.09, −0.03]	90 %
Zhou et al. (2022)	[−1.26, −0.08]	91 %

### *Publication bias*

Publication bias was evaluated utilizing Egger's test. The p*-*value obtained from Egger’s regression analysis was 0.683. No significant evidence of publication bias was detected in the trials assessing the effect of art therapy on reducing depression in children and adolescents.

## Discussion

The present study’s objective was to systematically quantify the evidence regarding the efficacy of art therapy-based interventions in mitigating depressive symptoms among children and adolescents. The findings imply that such interventions may outperform control conditions in diminishing depressive symptoms within this demographic. To the best of our knowledge, this is the first systematic review to examine the impact of art therapy on depressive symptoms in the children and adolescent population.

Subgroup analyses indicated that painting therapy, a modality within art therapy, exhibited potentially significant effects in alleviating depressive symptoms among children and adolescents. The high degree of heterogeneity observed across studies may be attributable to variations in study designs, sample characteristics, or the specific implementation of painting therapy interventions. The evidence for the efficacy of music therapy and dance therapy in treating depressive symptoms was less conclusive, necessitating further research to elucidate their potential roles in this context.

The findings of this systematic review suggest that art therapy interventions demonstrate broad applicability in alleviating depressive symptoms among children and adolescents across various disease contexts, including type 1 diabetes, obesity, social and emotional disorders, cancer, depression, and burn injuries. This cross-disease positive effect may be attributable to the unique capacity of art therapy to facilitate emotional release and self-exploration through creative expression, transcending the specific disease context^31^ Furthermore, the non-verbal modality of art therapy renders it particularly suitable for young individuals who may find it challenging to articulate their internal experiences^32^ However, it is essential for art therapists to exercise flexibility and sensitivity when addressing the distinct therapeutic challenges and psychosocial needs that arise from varying disease backgrounds^31^

The findings of this review are consistent with previous research, further corroborating the significant effects of art therapy in alleviating depressive symptoms and effectively reducing their occurrence among children and adolescents^33^ Notably, a recent innovative study by Nan et al^34^ evaluated treatment effects by analyzing cortisol levels in hair samples and found that clay-based art therapy can effectively modulate emotional states. Additionally, Blomdahl and Goulias,^11^ in assessing the feasibility and acceptability of art therapy interventions for depressed adolescents in child and adolescent psychiatry settings, also observed statistically significant improvements in depressive symptoms. Furthermore, a published systematic narrative review highlighted the responsiveness of art therapy, wherein therapists can flexibly employ various means and forms of expression and adjust therapeutic behaviors to meet clients' personalized needs and circumstances. This personalized adaptability is pivotal in achieving the positive psychosocial outcomes associated with art therapy^31^ It also accounts for the high heterogeneity often observed in meta-analyses of art therapy, as its application is customized to the unique circumstances of each participant.

The positive effects of art therapy as an intervention for depressive symptoms in children and adolescents may be mediated through multiple psychological and neurobiological mechanisms. First, according to the Expressive Therapies Continuum (ETC) model, art therapy provides individuals with a unique emotional and cognitive experience through its different levels of information processing, including kinesthetic/sensory, perceptual/affective, cognitive/symbolic, and creative levels^35^ This hierarchical approach facilitates the exploration of inner emotions and enhances emotional expression and self-understanding through the perceptual and cognitive activities involved in the art-making process^36^ Furthermore, the effects of art therapy are associated with the brain's Large-Scale Brain Networks (LSBNs), particularly the Salience Network (SN), which integrates emotional and cognitive information and is closely linked to communication, social behavior, and self-awareness^37^ Engagement in art therapy can augment the SN's activity, which aids in curbing self-criticism and enhancing focus on goal-directed activities^38^ Art therapists, through questioning and guidance, such as “What do you see, and how does it make you feel?”, can further facilitate the integration of perception and emotion in individuals, contributing to improved emotion regulation and self-awareness^35^ Moreover, another crucial mechanism of art therapy is its provision of a creative outlet, enabling children and adolescents to process and express complex emotions through non-verbal means, which may be challenging to achieve through traditional verbal therapy^32^ Additionally, the group setting of art therapy interventions provides opportunities for social interaction and support, which are instrumental in alleviating feelings of loneliness and enhancing social skills^39^ Based on the above analysis, it can be concluded that art therapy offers a comprehensive intervention approach for depressive symptoms in children and adolescents, facilitating emotional expression, cognitive integration, social interaction, and self-awareness development.

This meta-analysis is subject to several limitations. Firstly, the included studies employed diverse forms of art therapy, and the scoring systems for these different modalities may vary across studies; hence, the SMD was utilized to mitigate potential bias in the effect size estimates arising from these variations in art therapy forms. Secondly, the field of art therapy is characterized by a lack of trial standardization; the diverse modes of expression, such as painting, music, and dance, require further research to align the appropriate form of therapy with individual participant needs. Thirdly, the long-term efficacy of art therapy (lasting ≥ 8-weeks) has been assessed in a limited number of studies, precisely one, which restricts the ability to conclude its sustained impacts^23^ Lastly, the effectiveness of dance-based art therapy for depression has been reported in a solitary trial, precluding a comprehensive evaluation of its quantitative benefits.

## Conclusion

The present study’s findings support the efficacy of art therapy as an intervention for alleviating depressive symptoms in children and adolescents. Therefore, art therapists and psychotherapists may consider integrating easily accessible and non-threatening art therapy modalities into their therapeutic repertoire to help alleviate depressive symptoms in young patients. However, for a more accurate assessment of the effectiveness of art therapy as an intervention, future research should employ more rigorous study designs. Additionally, investigating the long-term impact and sustainability of art therapy interventions is an essential direction for future research, which will help further establish the position of art therapy in mental health treatment policies and guidelines.

## Authors’ contributions

Bo Zhang: Conceptualization; methodology; writing-original draft preparation.

Lifang Yang: Data curation; software; validation.

Wen Sun: Investigation; visualization.

Peng Xu: Formal analysis; writing-reviewing and editing.

Hui Ma: Supervision; project administration.

Azizah binti Abdullah: Writing-reviewing.

## Declaration of competing interest

The authors declare no conflicts of interest.
